# LHP1 Could Act as an Activator and a Repressor of Transcription in Plants

**DOI:** 10.3389/fpls.2017.02041

**Published:** 2017-11-28

**Authors:** Jing Feng, Jiang Lu

**Affiliations:** ^1^Guangxi Crop Genetic Improvement and Biotechnology Laboratory, Guangxi Academy of Agricultural Sciences, Nanning, China; ^2^Center for Viticulture and Enology, School of Agriculture and Biology, Shanghai Jiao Tong University, Shanghai, China

**Keywords:** polycomb group, LHP1, chromatin, histone modification, *Arabidopsis thaliana*

## Abstract

Polycomb group (PcG) proteins within the polycomb repressive complex 1 (PRC1) and PRC2 are significant epigenetic regulatory factors involved in important cellular and developmental processes in eukaryotes. In Arabidopsis, LIKE HETEROCHROMATIN PROTEIN 1 (LHP1), also known as TERMINAL FLOWER 2, has been proposed as a plant specific subunit of PRC1 that could bind the trimethylated lysine 27 of histone H3 (H3K27me3), which is established by PRC2 and is required for a functional plant PcG system. LHP1 not only interacts with PRC1 to catalyze monoubiquitination at lysine 119 of histone H2A but also functions with PRC2 to establish H3K27me3. This review is about the interaction of LHP1 with PRC1 and PRC2, in which LHP1 may act as a bridge between the two. Meantime, this review highlights that LHP1 could act as an activator and a repressor of transcription.

## Overview of PcG Protein, LHP1

Eukaryotic genomic DNA is packaged in the nucleus around histone octamers into nucleosomes forming the chromatin structure, consisting of DNA, RNA, histones and non-histone proteins. Each nucleosome contains a stretch of DNA wrapped around an octamer of histone proteins comprising two molecules each of the four core histones H2A, H2B, H3, and H4 (Luger et al., 1997). Each histone has a protrudent N-terminal and/or C-terminal tail and can be subjected to covalent modification by acetylation, methylation, phosphorylation, and other enzymatic reactions (Feng and Shen, 2014; Shi et al., 2014). Covalent modifications of DNA and histones play critical roles in regulating chromatin structure and gene expression, mainly by altering its compaction and shaping the three-dimensional topology of the genome (Mozgova and Hennig, 2015).

The evolutionarily conserved Polycomb group (PcG) proteins broadly found in many multicellular organisms participate in one of the earliest epigenetic regulatory mechanisms. They were originally identified in *Drosophila* spp. as repressors of homeotic genes (Lewis, 1978). PcG proteins form multiprotein Polycomb Repressive Complexes PRC1 and PRC2, which both have functions in the epigenetic repression of gene expression via histone modifications (Bemer and Grossniklaus, 2012; Molitor and Shen, 2013; Grossniklaus and Paro, 2014; Mozgova and Hennig, 2015; Xiao and Wagner, 2015). PRC2 catalyzes histone H3 lysine-27 trimethylation (H3K27me3), forming a docking site for PRC1, which subsequently recognizes/reads the H3K27me3 mark and further mediates deposition of downstream H2A monoubiquitination (H2Aub1) to repress gene expression (Simon and Kingston, 2013; Mozgova and Hennig, 2015; Xiao and Wagner, 2015). Accordingly, PRC1 activity was proposed to stabilize the repression of H3K27me3 marked loci. However, in recent years there have been numerous reports challenging this model. The relationships between PRC1 and PRC2 components may be far more complex than initially anticipated. PRC1 can be recruited independent of, or even prior to, H3K27me3 deposition, which indicates that PRC1 may act as a docking point for PRC2 and subsequent spreading of H3K27me3 marks at some target genes (Kim et al., 2012; Yang et al., 2013; Calonje, 2014; Molitor et al., 2014; Shen et al., 2014; Merini and Calonje, 2015). For example, during post-germinative repression of seed maturation genes, PRC1 acts upstream of PRC2, since H2Aub preceded H3K27me3 (Yang et al., 2013; Calonje, 2014; Molitor et al., 2014). To date, these possible mechanisms are unclear, especially in plants. In this review, we summarize the recent findings of the role of PcG protein, LHP1, during various developmental programs in plants and highlight that LHP1 could act as an activator and a repressor of transcription.

## LHP1 Gene and Protein Structure

Up to the present, many plant *LIKE HETEROCHROMATIN PROTEIN1* (*LHP1*) homologs have been identified (Zemach et al., 2006; Mimida et al., 2007; Exner et al., 2009; Hennig and Derkacheva, 2009). However, in addition to Arabidopsis, the current studies of LHP1 are limited to the differences in protein expression profile, but not in-depth study of gene expression, functions, and evolution of plant LHP1. This review, the focus has been on the character of LHP1 in Arabidopsis. *LHP1* was initially identified in screens for inflorescence meristem function and was named *TERMINAL FLOWER 2* (Larsson et al., 1998; Kotake et al., 2003). *LHP1* is a single-copy gene encoding a functional homolog of Pc, despite its structural homology with the protein of metazoan HETEROCHROMATIN PROTEIN 1 (HP1) (Gaudin et al., 2001; Kotake et al., 2003). The Arabidopsis *LHP1* gene that encodes an ∼45 kDa protein composed of 445 amino acids is composed of six exons and five introns and localized to chromosome 5 (AT5G17690). LHP1 protein structure is highly evolutionarily conserved and possesses significant homology to HP1, which contains two major structural domains: a chromo domain (CD) and a chromo shadow domain (CSD) (Gaudin et al., 2001; Libault et al., 2005; Zemach et al., 2006). However, HP1 is capable of binding to heterochromatic regions of the genome and binds to H3K9me2/3, whereas LHP1 usually localizes to euchromatin and is needed for maintenance of gene silencing in euchromatin, but not in heterochromatin, which has an affinity for H3K9me3 and H3K27me3 marks (Libault et al., 2005; Nakahigashi et al., 2005). The chromo domain is essential for ensuring the function of the LHP1 protein. Disruption in the functional chromo domain caused a phenotype similar to the *lhp1* null mutant and H3K27me3 could not be recognized (Exner et al., 2009). The CSD is also very important for the function of LHP1, which has a potential protein–protein interaction site (Cowieson et al., 2000). LHP1 possesses five K-R/K-X-R/K classical nuclear localization signals (NLSs) (Dingwall and Laskey, 1991). Four NLS are located in the N-terminal end of the protein, while one single motif is present in the C-terminal end.

## LHP1 As A Bridge Between PRC1 and PRC2

PRC2s are highly conserved between animals and plants (Chen and Rasmuson-Lestander, 2009; He et al., 2013). For example, the four PRC2 core components in Arabidopsis contain three homologs of the histone methyltransferase enhancer of zeste E(z), namely, CURLY LEAF (CLF), SWINGER (SWN), and MEDEA (MEA) (Grossniklaus et al., 1998; Chanvivattana et al., 2004; Schubert et al., 2006), three homologs of the suppressor of zeste: EMBRYONIC FLOWER 2 (EMF2), FERTILIZATION INDEPENDENT SEED 2 (FIS2) and VERNALIZATION 2 (VRN2) (Luo et al., 1999; Gendall et al., 2001; Yoshida et al., 2001), a single homolog of the *Drosophila* ESC WD-40 protein: FERTILIZATION INDEPENDENT ENDOSPERM (FIE) (Ohad et al., 1999), and five homologs of p55: MULTICOPY SUPPRESSOR OF IRA 1–5 (MSI1–5) (**Table 2**). Until now, only MSI1 has been demonstrated as part of the PRC2 complex (Hennig et al., 2005). In contrast, the existence of plant PRC1 complex was debated for a long time, and PRC1 compositions are drastically diverged in plants compared to animals (Molitor and Shen, 2013). In plants, PRC1 contains two RING1 homologs (AtRING1a and AtRING1b), three Psc/PCGF homologs (AtBMI1a, AtBMI1b, and AtBMI1c, Xu and Shen, 2008; Bratzel et al., 2010; Chen et al., 2010), and two other plant-specific proteins EMBRYONIC FLOWER1 (EMF1) and LHP1 (Gaudin et al., 2001; Calonje et al., 2008) (**Table 1**).

**Table 1:** PRC1 core proteins in model species.

*Drosophila*	Human	Arabidopsis
Pc (polycomb)	CBX2/HPC1	LHP1/TFL2
	CBX4/HPC2	
	CBX6	
	CBX7	
	CBX8/HPC3	
Ph (polyhomeotic)	HPH1/EDR1/PHC1	
	HPH2/EDR2/PHC2	
	HPH3/EDR3/PHC3	
Psc (posterior sex combs)	PCGF1/RNF68/NSPC1	AtBMlla
	PCGF2/MEL18/RNF110	AtBMllb
	PCGF3/RNF3	AtBMllc
	PCGF4/BMI1/RNF51	
	PCGF5/RNF19	
	PCGF6/MBLR/RNF134	
Sce (sex combs extra)/dRing	Ringl/RINGla/RNFl	AtRINGla
	Ring2/RINGlb/RNF2	AtRINGlb


**Table 2:** PRC2 core proteins in model species.

*Drosophila*	Human	Arabidopsis
E(z) (enhancer of zeste)	EZH1	CLF
	EZH2	MEA
		SWN
Su(z)12 (suppressor of zeste)	Su(z)12	FIS2
		EMF2
		VRN2
Esc (extra sex combs)	EED	FIE
P55	RbAp45/48	MSI1-5


Yeast two-hybrid and pull-down assays showed that AtRING1a, AtBMI1a, AtBMI1b, and AtBMI1c directly interact with LHP1 (Xu and Shen, 2008; Bratzel et al., 2010; Chen et al., 2010). The PRC1 component EMF1 also directly interacts with LHP1 (Wang et al., 2014), indicating that LHP1 is a core component of PRC1 and can be present in several PRC1-like complexes in Arabidopsis. In addition, genetic evidence also demonstrated physical interactions between AtRING1 and LHP1. The single *lhp1^-/-^* mutant shows pleiotropic phenotypes, including small, narrow and upward-curled rosette leaves, reduced plant height (more than 50% reduction), early flowering, a terminal flower structure, and smaller and fewer siliques (Larsson et al., 1998; Gaudin et al., 2001; Kotake et al., 2003). *Atring1a^-/-^Atring1b^-/-^lhp1^-/-^* mutants have more pronounced phenotypes: the plants are extremely small with very few sessile leaves and completely arrested inflorescences (Xu and Shen, 2008). In addition, a recent report showed that LHP1 co-purifies with PRC2, and LHP1 can directly bind MSI1 *in vitro* and can be co-immunoprecipitated with MSI1 and EMF2 *in vivo*, showing a complex interaction between LHP1 and EMF–PRC2. The LHP1–PRC2 interactions are, therefore, likely facilitating recruitment of PRC2 to target genes and LHP1 and PRC2 are closely integrated in Arabidopsis (Derkacheva et al., 2013; Wang et al., 2016). LHP1 may act as a bridge between PRC1 and PRC2 as it could directly interact with members of the two.

## Role of LHP1 in Flowering Time Regulation

Accurate flowering time is critical for the reproductive success of plants. FLOWERING LOCUS T (FT) plays a central role in promoting flowering, and the leaf vein is the site where various flowering-regulatory genes converge to regulate FT mRNA expression (Turck et al., 2008). Compared to wild-type, loss-of-function *lhp1* mutants exhibit a photoperiod-independent early flowering phenotype. Overexpression of LHP1 (35S:LHP1) showed little effect on flowering time, and *ft* mutations completely suppressed the early flowering of *lhp1*. Molecular analyses of the mutants indicated that LHP1 controls flowering time primarily by recognizing and binding to H3K27me3 and directly interacts with FT chromatin repression of *FT* expression. Studies have demonstrated a high correlation between LHP1 and H3K27me3 deposition, and in the *lhp1* background, H3K27me3 deposition was globally reduced (Turck et al., 2007; Veluchamy et al., 2016). However, increasing LHP1 does not delay the timing of floral transition in wild-type Arabidopsis (Takada and Goto, 2003). LHP1 mRNA is widely expressed in all wild-type tissues, but in mature leaves, LHP1 mRNA is only expressed in the vascular tissue (Kotake et al., 2003; Takada and Goto, 2003). EMF1, LHP1, and a histone H3 lysine-4 demethylase form a distinct PcG complex, termed EMF1c, directly represses the *FT* expression in the vascular tissue before dusk and at night (Wang et al., 2014). In the long-day pathway, CONSTANS (CO) directly activates *FT* expression and disrupts EMF1 binding to FT chromatin in leaf veins, specifically at dusk (Takada and Goto, 2003). EMF1-PcG complex plays a role downstream of the photoperiod pathway to control FT expression in the phloem. The vascular EMF1c complex integrates several flowering-regulatory pathways to synchronize flowering time to environmental cues. In addition, in vernalization and the autonomous pathway, LHP1 is part of a multiprotein complex with PRC2 to re-establish H3K27me3 in the repression of *FLC* expression.

## Role of LHP1 in Root Development

Roots are essential organs for absorption, utilization of water and mineral nutrients. The phytohormone auxin plays vital roles for successful growth and development of plants. Altered auxin levels rapidly induce changes in gene expression and cause a change in phenotype or function. In the *lhp1* mutant, auxin levels are lower than in wild-type. To investigate the mechanism, the auxin biosynthetic pathways have been studied. The results showed that the expression of auxin biosynthesis related *YUCCA* genes are down-regulated, resulting in low levels of auxin in the *lhp1* mutant. Moreover, LHP1 was found to directly regulates genes in the auxin biosynthetic pathway (Rizzardi et al., 2011). This suggests that LHP1 is involved in auxin biosynthesis through positive regulation of *YUCCA* genes. In the Arabidopsis root, SCARECROW (SCR), of a transcription factor of plant-specific GRAS family, is required for the first cell division in the roots. The *lhp1* mutant has a premature middle cortex phenotype, which is similar to the *scr* mutant. ChIP-PCR was performed with LHP1 and SCR and demonstrated that LHP1 and SCR act together to suppress premature middle cortex formation (Cui and Benfey, 2009). Previous research showed that the phytohormone gibberellin (GA) plays a key role in middle cortex formation and in the *scr* mutant. GA suppressed the premature middle cortex phenotype, but the GA biosynthesis inhibitor paclobutrazol (Pac) enhanced the premature middle cortex phenotype (Paquette and Benfey, 2005). Comparing the *lhp1* mutant and wild-type roots after treatment with GA or with Pac, middle cortex formation was completely suppressed by GA and Pac caused middle cortex formation in the *lhp1* mutants but in none of the wild-type roots (Cui and Benfey, 2009). These results indicated that LHP1 and SCR suppress premature middle cortex formation by a similar mechanism, in which GA has a dominant role.

## Dual Role of LHP1 in Transcriptional Regulation

Generally, polycomb group proteins have been known as transcriptional repressors, which play crucial roles in the maintenance of the transcriptionally silenced state for proper cell differentiation in animals and plants. However, recent studies showed that LHP1 could also be an activator of transcription by differential expression analysis of LHP1-target genes in *lhp1* mutants versus wild-types (Veluchamy et al., 2016). By comparing H3K27me3 and RNA Pol II ChIP-Seq results, LHP1 and H3K27me3 deposition are highly positively correlated while LHP1 and RNA Pol II occupancy are negatively correlated. Furthermore, in the *lhp1* mutant, it was found that the majority of LHP1 targets were hypo-methylated. These results were consistent with the function of LHP1 as a transcriptional repressor. In addition, more than one-third of LHP1 disturbed target genes were down-regulated in the *lhp1* mutant, suggesting that LHP1 can function as a positive regulator of a set of genes (Veluchamy et al., 2016). Rizzardi et al. (2011) research showed that LHP1 exerts a positive role in transcriptional activation of the *YUCCA* genes.

## Role of LHP1 in Other Processes

LHP1 interacts with different proteins in different cell types to perform distinct functions, and to date, several proteins interacting with LHP1 in Arabidopsis have been identified. For example, it has been confirmed by yeast two hybridization, pull down and BiFc methods, that LHP1 directly interacts with leaf development regulators ASYMMETRIC LEAVES 1 (AS1) and AS2 *in vitro* and *in vivo*. The wild-type Col-0, *as1-1*, *as2-1*, and *lhp1* single mutants produced smaller rosette leaves, while the *as1-1 lhp1* and *as2-1 lhp1* double mutants produced much smaller rosette leaves (Li et al., 2016). This genetic evidence also showed that there is a interaction between LHP1 and AS1/AS2. Beside the direct interaction between AS1/AS2 and LHP, SHORT VEGETATIVE PHASE (SVP), a MADS box transcription factor, can recruit LHP1 to the SEPALLATA 3 (SEP3) gene and repress *SEP3* transcription, consequently preventing premature differentiation of floral meristems (Liu et al., 2009). Enhancer of LHP1 (*Eol1*), a gene related to yeast *Chromosome transmission fidelity 4* (*Ctf4*), recruits LHP1-PRC2 to maintain H3K27me3 levels in dividing cells in Arabidopsis (Zhou et al., 2017b). CYCLOPHILIN71 (CYP71), which controls leaf differentiation and leaf shape establishment, also has been identified to interact with LHP1, and both the chromo and CSDs in LHP1 are required for the interaction (Li and Luan, 2011). Loss of *AtCYP71* function results in dwarfism and distorted, small leaves, and delayed overall development. In addition, the double mutant, *lhp1 cyp71*, showed more severe phenotypes than the single mutants. This genetic evidence indicates that CYP71 and LHP1 synergistically control plant development (Li and Luan, 2011). In addition, in the context of DNA replication, LHP1 interacts with two DNA polymerase (pol) catalytic subunits, INCURVATA2 (ICU2, a subunit of DNA pol alpha) and EARLY IN SHORT DAYS 7 (ESD7, a subunit of DNA pol epsilon) (Barrero et al., 2007; del Olmo et al., 2010). *In vitro*, LHP1 was reported to interact with the GAGA factor BASIC PENTACYSTEINE6 (BPC6) and was recruited to *GAGA* motifs (Hecker et al., 2015). The most recent research indicates that LHP1-INTERACTING FACTOR2 (LIF2), with three RNA-recognition motifs (RRMs), is involved in Arabidopsis thaliana cell fate and stress responses and has been shown to interact with LHP1 mediated by the conserved CSD. Interestingly, this interaction links chromatin dynamics, RNA processing, and developmental plasticity together (Latrasse et al., 2011; Molitor et al., 2016). There is evidence that LHP1 interacts with the APOLO long non-coding RNA during the dynamic regulation of chromatin three-dimensional conformation in response to auxin (Ariel et al., 2014). Veluchamy et al. (2016) showed that LHP1 can play a role in the modulation of global chromatin architecture as genome topology was globally altered in the *lhp1* background.

## Conclusion

In summary, these data strongly suggest that the roles of LHP1 are more complex than once previously thought. LHP1 interacts with several proteins in several pathways to perform its distinct functions. Firstly, it recognizes H3K27me3 in a classic PcG-mediated transcriptional repression pathway. Secondly, transcription factors interact with LHP1 and recruit it to specific DNA elements to initiate gene silencing. Thirdly, through interacting with a replication complex, LHP1 possibly form a high-order complex with polymerase to recruit other PRC2 components to targets after DNA replication. However, more recent research findings argue against the classic PcG-mediated transcriptional repression pathway. For example, LHP1 was originally proposed to be the counterpart of Pc, responsible for recruitment of PRC1 to H3K27me3 catalyzed by PRC2, but recent studies showed that H3K27me3 modifications also require LHP1 (Liu et al., 2009; Derkacheva et al., 2013) and LHP1 coordinates with CLF to regulate H3K27me3 modification and target gene repression. In addition, both CLF and LHP1 are involved in spreading of H3K27me3 marks (Wang et al., 2016). In the classic PcG pathway, PRC2-mediated H3K27me3 recruits PRC1 that in turn monoubiquitinates H2A. Recent study, however, showed that LHP1 is not required for H2AK121ub marking and PRC2 activity is dispensable to establish H2AK121ub marks at most genes (Zhou et al., 2017a). It is obvious that in PRC1-mediated gene repression, the roles of these LHP1 interacting proteins have yet been fully understood. In addition, it is still unclear that in which context they are recruited to specific genes to carry out their functions. Moreover, only a few LHP1 interacting proteins have been identified. Thus, further in depth researches are needed to identify more LHP1-binding proteins and their functions.

Generally, LHP1 is known to act as a transcriptional repressor, both in plants and animals. Recently, growing evidences show that LHP1 can also act as a activator. For example, LHP1 positive regulates *YUCC4* expression (Rizzardi et al., 2011), LHP1 exerts a positive role in environmental stimuli (Veluchamy et al., 2016) and LHP1 interacts with GmPHD6 to form a transcriptional activation complex to regulate genes for salt tolerance (Wei et al., 2017). At present, although the study of plant LHP1 as an activator is limited, studies have long been shown that HP, a homologs of LHP1, can act as activators. For example, HP1 has been reported involvement in the activation of several euchromatic genes in *Drosophila*. By immunostaining experiments using an HP1 antibody, it found that HP1 is associated with developmental and heat shock-induced puffs on polytene chromosomes. And HP1 is positively involved in *Hsp70* gene activity (Piacentini et al., 2003). Mammalian HP1 homologs together with dimethylated H3K9 also associate with many active transcription units (Vakoc et al., 2005). In addition, the mammalian HP1γ and *Drosophila melanogaster* HP1α, involving in positive euchromatic gene regulation (Piacentini et al., 2003; Cryderman et al., 2005; Vakoc et al., 2005; Fanti and Pimpinelli, 2008; Smallwood et al., 2012; Eissenberg and Elgin, 2014). In *Drosophila*, HP1α positively regulates euchromatic gene expression through RNA transcript association and interaction with hnRNPs (Piacentini et al., 2003, 2009; De Lucia et al., 2005; Vermaak and Malik, 2009; Piacentini and Pimpinelli, 2010). HP1c, a homolog of HP1, appears to be linked to transcription activity rather than repression in *Drosophila* (De Wit et al., 2007). Regardless of the positive and negative effects of HP1, are determined by its interacting proteins. The activator and repressor activities require distinct protein domains for different DNA–protein, RNA–protein, or protein–protein interactions. HP1 has different domains that should confer it to the necessary structural flexibility required for multiple functional roles. Plant *LHP1* is able to perform several of the functions carried out by the *HETEROCHROMATIN PROTEIN1* family in animals. However, the molecular mechanisms underlying its dual role on gene expression remain to be elucidated.

## Author Contributions

JF wrote this manuscript and JL provide assistance.

## Conflict of Interest Statement

The authors declare that the research was conducted in the absence of any commercial or financial relationships that could be construed as a potential conflict of interest.
